# Cultivar-Specific Performance and Qualitative Descriptors for Butterhead Salanova Lettuce Produced in Closed Soilless Cultivation as a Candidate Salad Crop for Human Life Support in Space

**DOI:** 10.3390/life9030061

**Published:** 2019-07-14

**Authors:** Christophe El-Nakhel, Maria Giordano, Antonio Pannico, Petronia Carillo, Giovanna Marta Fusco, Stefania De Pascale, Youssef Rouphael

**Affiliations:** 1 Department of Agricultural Sciences, University of Naples Federico II, 80055 Portici, Italy; 2 Department of Environmental, Biological and Pharmaceutical Sciences and Technologies, University of Campania “Luigi Vanvitelli”, 81100 Caserta, Italy

**Keywords:** antioxidant molecules, BLSS, daily water uptake, functional quality, *Lactuca sativa* L., mineral profile, physiological parameters, space farm, water use efficiency

## Abstract

Plant production is crucial for space journeys self-autonomy by contributing to the dietary intake necessary to sustain the physical and psychological well-being of space colonists, as well as for contributing to atmospheric revitalization, water purification and waste product recycling. Choosing the appropriate cultivar is equally important as the species selection, since cultivar influences the obtained fresh biomass, water use efficiency (WUE), growing cycle duration, qualitative features and postharvest performance. Two differently pigmented butterhead *Lactuca sativa* L. (red and green Salanova) cultivars were assessed in terms of morphometric, mineral, bioactive and physiological parameters. The experiment was carried out in a controlled environment growth chamber using a closed soilless system (nutrient film technique). Red Salanova registered a biomass of 130 g at harvest, which was 22.1% greater than green Salanova, and a water uptake of 1.42 L during the full growing period corresponding to WUE of 91.9 g L^−1^, which was 13.8% higher than that of green Salanova. At harvest, green Salanova had accumulated more P, K, Ca, Mg and 37.2% more nitrate than red Salanova, which however had higher relative water content, leaf total and osmotic potential and higher SPAD index. Red Salanova also exhibited at harvest around two-fold higher lipophilic antioxidant activity and total phenols, and around six-fold higher total ascorbic acid levels. These latter characteristics improved the antioxidant capacity of red Salanova enabling it to use light more efficiently and deliver better overall performance and yield than green Salanova. Moreover, the higher phenolics and total ascorbic acid contents of red Salanova constitute natural sources of antioxidants for enriching the human diet and render it an optimal candidate cultivar for near-term missions.

## 1. Introduction

When humans rove far from Earth orbit, horticulture will doggedly follow [1]. Deep space voyages cannot lean on conveyance from Earth, this umbilical reliance and replenishment will not be an option anymore [2]. Therefore, in order to extend space journeys, humans during their missions should be able to provide proper dietary intake [3], by being self-sufficient and producing fresh food that is crucial for retaining physical [4] and psychological well-being [4,5]. A plant-food-based diet is premium to nourish body and soul [6], making sustainable plant production in space a primary objective of research activities [7]. Therefore, in order to support numerous crew members for long-duration space missions Bio-regenerative Life-Support Systems (BLSS) have been designed to eventually eliminate the need to rely on resupply from Earth [4]. A life support system is pivotal for regenerating all survival essentials [7]. In it, higher plants play an essential role, as atmosphere revitalizer through CO_2_ absorption and O_2_ emission, water purifier through transpiration [2,3,5,7,8] and organic wastes recycler via mineral nutrition [7].

Higher plants growth chamber in BLSS denote the compartment IVb. It stands for a paramount mantle in MELiSSA’s (Micro-Ecological Life Support System Alternative) loop. This latter aims the fulfilment of a viable ecological ‘niche’ for humans in the outer space, still functional notwithstanding the utter disconnection from the Earth, focusing on the interaction of the organism within its environment as unit of life [2]. An appropriate selection of the species (crop) for this compartment can supply food as portion of the produced biomass [8] and provide a myriad of nutrients including biologically active compounds with antioxidant, antibacterial and antiviral effects able to stimulate the immune system [6]. The main shared criteria through which the candidate cultures for the space are selected are: broad nutritional coverage, harvest index, crop efficiency and potential yield [3,7,8,9]. Namely, salad crops have a very high harvest index, low water uptake/transpiration ratio, brief growing cycle and require little crew attention to be grown [3]. 

Lettuce was nearly omnipresent in crops list suggested or studied for life support systems as candidate “salad” crops (i.e., tomato, radish, lettuce, spinach, chard, and carrot) for near-term missions [1]. Moreover, it topped chart scores of space/time efficiency, harvest index, light/energy use efficiency and handling time, as well as scoring the highest among selected crops to be cultivated in the Future Exploration Greenhouse (FEG) at Neumayer Station III and in the International Standard Payload Rack (ISPR) on the International Space Station (ISS) [4]. Nevertheless, even cultivars of candidate crops undergo a series of selection to choose the appropriate ones [9]. Moreover, lettuce nutrient composition and bioactive compounds vary among type and pigmentation as well [10] which can influence the selection. On the other hand, water and nutrient management are demanding features for plant cultivation in life support systems. Therefore, recirculating hydroponic systems are favoured [7] to remove water and nutrient stress, improve production, obtain higher water use and dispense less nutrients [8], leading to an effective resource management [11]. Such inputs emphasize on the importance of a continuous ground experiment in order to monitor lettuce water absorption during a growing cycle in a closed loop hydroponic system. To our knowledge, no previous work has focused on measuring butterhead Salanova lettuce water uptake, physiological and qualitative aspects on three-days-interval basis and covering the full growing period.

Based on this approach, the purpose of this paper was to elaborate the evolution of two differently pigmented butterhead lettuce regarding water uptake, morphological, physiological and qualitative data through a complete nineteen-days-growing period. The experiment was carried out in a Fitotron growth chamber in a closed soilless system of nutrient film technique (NFT). The gained data can be appreciated by space-faring colonists in order to know in advance the water consumption of butterhead lettuce cultivar Salanova, nutrient accumulation and detecting the adequate maturity stage for harvesting in order to maintain optimal quality in storage, and more importantly these findings are appreciated by terrestrial controlled environment agriculture.

## 2. Materials and Methods 

### 2.1. Plant Material, Growth Chamber Conditions, Experimental Design and Harvesting Schedule

Two butterhead lettuce cultivars (*Lactuca sativa* L. var. *capitata*) green Salanova® and red Salanova® (Rijk Zwaan, Der Lier, The Netherlands) were cultivated for 19 days in a controlled closed soilless system. The experiment was carried out in a 28 m^2^ open-gas-exchange growth chamber (7.0 m × 2.1 m × 4 m; W × H × D), at the Department of Agricultural Sciences of the University of Naples Federico II, Italy. Lettuce plants were cultivated in a nutrient film technique (NFT) growing system, consisting of propylene gullies covered with white polyethylene film to avoid evaporation of the nutrient solution (NS) and to reflect the incident light. The gullies were 200 cm long, 14.5 cm wide and 8 cm deep each, having a sloping degree of 1%. The NS was delivered by submerged pumps at a constant flow of 1.5 L min^−1^ and then was collected in 25 L polypropylene tanks by gravity dependent flow. The NS consisted of a modified Hoagland and Arnon formulation: 9.0 mM N-NO_3_^−^, 2.0 mM S, 1.0 mM P, 4.0 mM K, 4.0 mM Ca, 1.0 mM Mg, 1.0 mM NH_4_^+^, 15 µM Fe, 9 µM Mn, 0.3 µM Cu, 1.6 µM Zn, 20 µM B, and 0.3 µM Mo. The electrical conductivity (EC) of the nutrient solution was 1.5 dS m^−1^, while the pH of the NS was monitored daily and maintained at 6.0 ± 0.2. Seeds of lettuce were germinated in vermiculite. Lettuce seedlings were transplanted 15 days after sowing, at two-true leaf stage in rockwool cubes (7 × 7 × 7 cm) (Delta, Grodan, Roermond, The Netherlands) placed into the gullies with an intra-row spacing of 15 cm and an inter-row spacing of 43 cm, making a density of 15.5 plants per square meter.

Light was supplied by high-pressure sodium lamps, with an intensity of 420 µmol m^−2^ s^−1^(165 cm from the top of the canopy) according to a light/dark regime of 12/12 h with corresponding temperature and relative humidity (RH) of 24/18 °C and 60/80%, respectively, the latter being maintained by a fog system. The experiment was carried out at ambient CO_2_ concentration (370–410 ppm) and air circulation and dehumidification were guaranteed by two heating, ventilation and air conditioning systems (Spagnol srl Greenhouse Technologies, Treviso, Italy).

Treatments of the two butterhead Salanova cultivars were arranged in a randomized complete-block design with 3 replicates, making a total of 216 plants divided in 36 experimental units made of six plants each. Treatments were six harvests separated by a three-day interval, starting at four days after transplant (DAT) and ending at 19 DAT. All measurements and analysis where executed at each harvest. 

### 2.2. Sampling, Growth Analysis, and SPAD Index Measurement

Plants were sampled six times during the crop cycle at 4, 7, 10, 13, 16 and 19 DAT, noting that at 1 DAT part of the seedlings was harvested. At each date, harvested plants were in a part frozen in liquid nitrogen and stored at −80 °C for qualitative analysis, and in a part used for biometric measurements, such as leaf number, fresh weight and leaf area, the latter being measured by an Area Meter (LI-COR 3100C biosciences, Lincoln, NE, USA). The Soil Plant Analysis Development (SPAD) index was measured on young healthy leaves by means of a portable chlorophyll meter SPAD-502 (Konica-Minolta, Tokyo, Japan) of three representative plants per experimental unit. Measurements were averaged to a single SPAD value per each replicate.

### 2.3. Water Uptake, Water Use Efficiency and Relative Growth Rate

Water level of all the tanks was measured on a three-day-basis interval, in order to detect water uptake evolution of the plants through the 19 days growing period. Then this volume was divided by three (number of days) and then by the number of plants per gully in order to express daily water uptake in mL plant^−1^ day^−1^, while cumulative water uptake was expressed in litres per plant. Water use efficiency (WUE) was calculated by dividing fresh yield of the plant by the volume of consumed water, and expressed in grams of fresh yield per litre. While relative growth rate (RGR) was calculated based on the following formula: RGR = (logeW_2_ − logeW_1_) / (t_2_ − t_1_), where W is the leaf dry matter and t is the sampling date.

### 2.4. Water Potential and Relative Water Content 

Leaf total water potential (Ψ_tot_) was measured on 4 cm leaf discs punched from young fully expanded leaves using a dew point potentiometer (WP4C, Decagon Devices, Pullman, WA, USA). Leaf osmotic potential (Ψ_π_) was measured after freezing and thawing leaf discs, while turgor pressure or pressure potential (Ψ_p_) was estimated as the difference between Ψ_tot_ and Ψ_π_, assuming that the matric potential is equal to zero. Leaf relative water content (RWC) was measured based on Colla et al. [12] with slight modifications. Briefly, each repetition consisted of 10 discs of 8 mm each, which were excised from the interveinal areas and weighed to determine fresh weight (FW), then floated in distilled water for 12 h to retrieve turgidity and re-weighted to determine turgid weight (TW). Finally, samples were dried at 80 °C for 48 h to determine dry weight (DW). RWC was calculated based on the following formula: RWC% = ((FW − DW)/ (TW − DW)) × 100. 

### 2.5. Dry Matter, Total Nitrogen and Mineral Content Analysis

Green and red butterhead Salanova lettuce were oven dried at 70 °C for three days, until reaching a constant weight, and then weighed again to determine the dry biomass on an analytical balance (Denver Instruments, Denver, CO, USA), and finally, dry matter (DM) percentage was calculated as DM = 100 × Dry weight/Fresh weight. Red and green Salanova leaf samples were ground separately in a Wiley Mill (Model 4, Thomas Scientific, Swedesboro, NJ, USA) to pass through a 841 microns screen, then 250 mg of the ground tissues were analysed by ion chromatography for mineral content: N, P, K, Ca, Mg and S as described in details by Rouphael et al. [13], mineral content was expressed in mg per g of dry weight. As for nitrate, it was expressed in mg per kg of fw according to the dry matter percentage of each sample. While total nitrogen was determined on one g of dried samples by the Kjeldahl method [14].

### 2.6. Analysis of Lipophilic Antioxidant Activity

Lipophilic antioxidant activity (LAA) was determined by using a radical cation assay, extracting 200 mg of lyophilized material by methanol. Based on Pelligrini et al. [15], 2,2’-azinobis 3-ethylbenzothiazoline-6-sulfonic acid (ABTS) method was used to measure LAA. The principle of the assay is that the inhibitory response of the radical cation is proportional to the antioxidant concentration and the reaction is complete at the time point selected of 2.5 min. A UV–VIS spectrophotometer was used to measure the absorbance reduction of the solutions at 734 nm wavelength to determine LAA. Results were expressed as mmol Trolox (6-hydroxy-2,5,7,8-tetramethylchroman- 2-carboxylic acid) per 100 g dw [15].

### 2.7. Analysis of Total Ascorbic Acid and Total Phenols

Total ascorbic acid (TAA) which is the sum of ascorbic acid (AA) and dehydroascorbic (DHA) was assessed by spectrophotometric detection on fresh plant tissues. DHA is reduced to AA by pre-incubation of the sample with dithiothreitol [16]. Quantification was performed by UV–VIS spectrophotometry (Hach DR 2000; Hach Co., Loveland, CO, USA) at 525 nm and the results were expressed as mg AA 100 g^−1^ fw.

Total phenolic content was determined in 60% methanol/water (*w*/*v*) extracts, according to the Folin-Ciocalteu procedure [17] using gallic acid as standard. Then 100 μL of the supernatant was combined with 400 μL of 7.5% sodium carbonate/water (*w*/*v*), samples were shaken for 15 min and then incubated for 30 min at room temperature. Absorption was measured at 765 nm using a UV–VIS spectrophotometer, and the results were expressed as mg gallic ac. eq. 100 g^−1^ dw.

### 2.8. Statistical Analysis

The Shapiro–Wilk and Kolmororov–Smirnov procedures were performed to verify that the data had a normal distribution, and the Levene, O’Brien and Bartlet tests were conducted to verify the homogeneity of variances. The obtained data were subjected to analysis of variance (ANOVA) using the software package SPSS 13 for Windows 2001. Means comparison between the DAT was performed with the use of Duncan’s Multiple Range Test (DMRT) at *p* ≤ 0.05. Moreover, comparison between the two cultivars was done using t-test. Regression analyses were performed on the mean values of the variables plotted for cultivar and DAT using SigmaPlot 12 software (Systat Software Inc., San Jose, CA, USA). The principal component analysis (PCA) was assessed using Minitab 16.8 statistical software [18,19]. The score plot and loading matrix were determined based on the first and second principal components (PCs). 

## 3. Results

### 3.1. Growth Response, Water Uptake and Water Use Efficiency 

**Figure 1:**
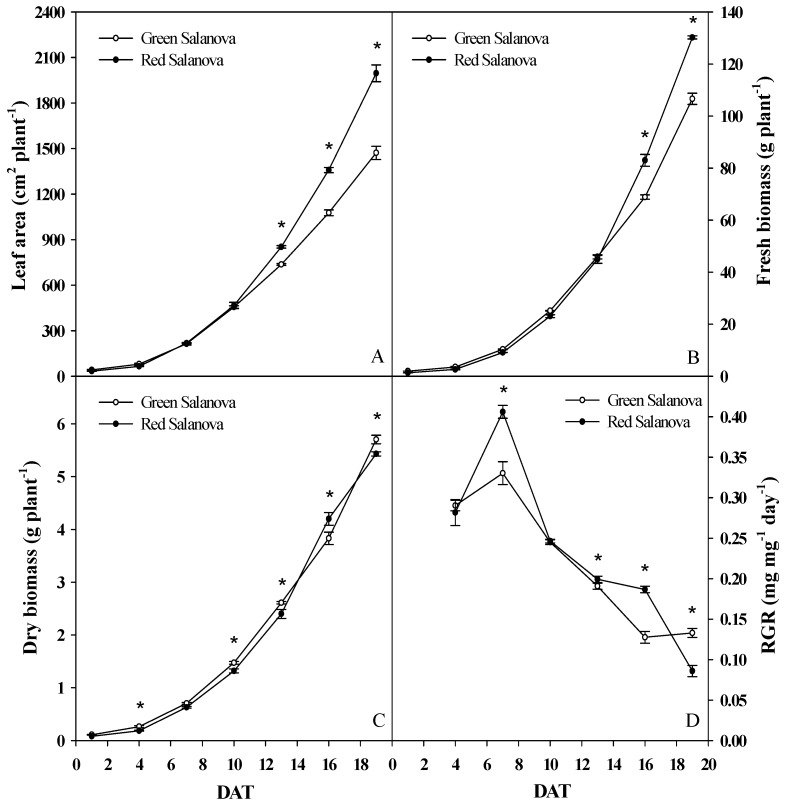
Evolution of leaf area (**A**), fresh biomass (**B**), dry biomass (**C**) and relative growth rate (RGR) (**D**) of red and green butterhead Salanova during the growing period. The values are means of three replicates. Asterisks indicate a significant difference at *p* ≤ 0.05 between cultivars.

Leaf area, fresh and dry biomass of both cultivars red and green Salanova showed an exponential increase during the 19 days growing period (Figure 1). A significant higher increase of red Salanova leaf area in comparison to green Salanova was obvious since 13 DAT and increased even more until 19 DAT, marking almost a 35.6% higher value. This is explained by the significant difference in the daily leaf area increase that is dominated by the red cultivar since 13 DAT (Table 1). Fresh biomass exhibited a significant difference between cultivars from 16 to 19 DAT for red Salanova, with a yield of 130.2 g plant^−1^, 22.1% larger than green Salanova that registered 106.6 g plant^−1^ at harvesting (Figure 1B). Both cultivars had no significant difference in terms of fresh biomass daily increase until 13 DAT, while during the subsequent growth period red Salanova showed significant higher daily increase (Table 1). As for dry biomass, it was significantly higher for the green cultivar almost in all the growing cycle, except around 16 DAT where red Salanova exhibited a significant higher increment (Figure 1C), which is evidenced in the daily changes presented in details in Table 1 with an increase of 0.6 g plant^−1^ day^−1^ for red Salanova in comparison to 0.41 g plant^−1^ day^−1^ for green Salanova. Dry biomass at harvest was 5.4 and 5.7 g plant^−1^ for red and green Salanova, respectively.

Considering Figure 1D, both cultivars showed an increase in RGR until 7 DAT, where red Salanova registered 0.41 mg mg^−1^ day^−1^, 24.2% greater than the green cultivar. After that, RGR of both cultivars decreased gradually over the growing period, with red being significantly higher from 13 to 16 DAT, but significantly decreasing less than the green cultivar towards 19 DAT and reaching 0.09 mg mg^−1^ day^−1^ at harvest, instead the green cultivar showed a slight increase of RGR at the end of the growing period and reached 0.13 mg mg^−1^ day^−1^.

**Figure 2:**
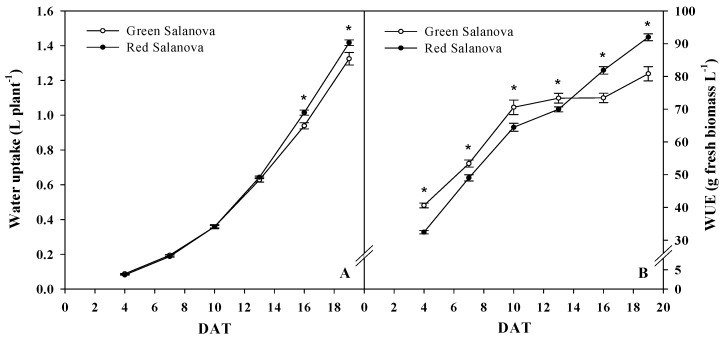
Evolution of water uptake (**A**) and water use efficiency (WUE) (**B**) of red and green butterhead Salanova during the growing period. Data are means of three replicates. Asterisks indicate a significant difference at *p* ≤ 0.05 between cultivars. Vertical bars indicate ± S.E. of the means, their absence indicates that the size was less than the symbol.

Cumulative water uptake, as well, demonstrated an exponential increase along the growing period as illustrated in Figure 2A, with a significant higher uptake noted for red Salanova starting at 16 until 19 DAT. This increase was clarified in Table 1, where a significant 19.2% higher increase of daily water uptake was registered at 16 DAT for the red cultivar, while no significant difference was mentioned for the rest of the growing period. Total water uptake at the end of the growing period registered 1.42 and 1.32 L plant^−1^ for red and green Salanova, respectively. As follows from Figure 2B, WUE displayed a quick increase the first 10 days after transplanting, then slowed down for green Salanova and formed a type of plateau between 10 and 16 DAT, while red Salanova WUE had a continuous increase all along the growing period, with significant higher values starting at 14 DAT in comparison to green Salanova that had significant higher WUE from transplant until 14 DAT. At the end of the growing period, red Salanova had a WUE of 92 g fresh biomass L^−1^, 13.8% higher than green Salanova WUE that registered 80.8 g fresh biomass L^−1^.

**Figure 3:**
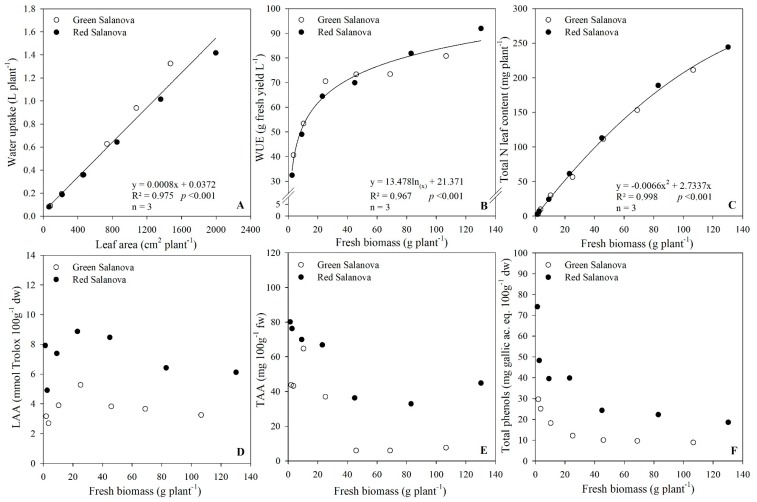
Relationship between water uptake and leaf area (**A**), water use efficiency (WUE) and fresh biomass (**B**), total N leaf content and fresh biomass (**C**), lipophilic antioxidant activity (LAA) and fresh biomass (**D**), Total ascorbic acid (TAA) and fresh biomass (**E**), total phenols and fresh biomass (**F**) for red and green butterhead Salanova during the growing period. Model equations and R^2^ values are presented when the fitness of the model was statistically significant (*p* < 0.001).

Mean daily increase of leaf area, fresh biomass and dry biomass was significantly higher for red Salanova, while there was no significant difference in water uptake between the two cultivars, which can explain why the red cultivar had higher WUE at harvesting (Table 1). Water uptake (L plant^−1^) was linearly correlated with leaf area (cm^2^ plant^−1^) (r = 0.99; *p* < 0.001), while fresh biomass (g plant^−1^) was positively correlated with WUE (r = 0.88; *p* < 0.001), as shown in Figure 3A,B for both cultivars with r^2^ = 0.967.

### 3.2. Leaf Macro-Mineral Composition 

**Figure 4:**
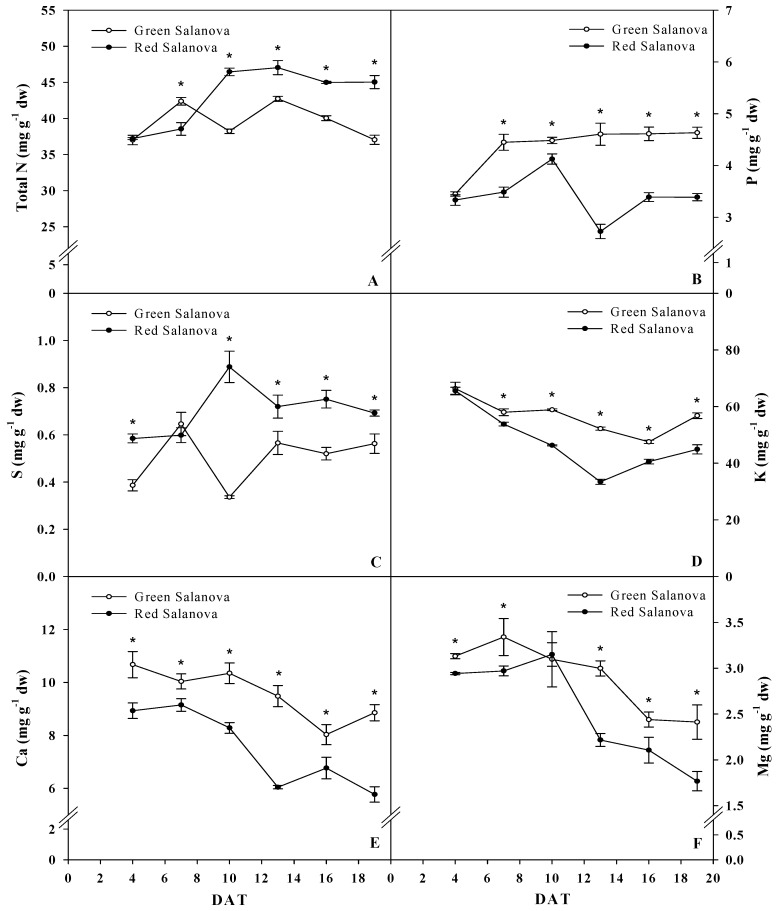
Evolution of total N (**A**), phosphorus (**B**), sulfur (**C**), potassium (**D**), calcium (**E**) and magnesium (**F**) concentrations of red and green butterhead Salanova during the growing period. Data are means of three replicates. Asterisks indicate a significant difference at *p* ≤ 0.05 between cultivars. Vertical bars indicate ± S.E. of the means, their absence indicates that the size was less than the symbol.

Figure 4 depicts leaf mineral concentration varying throughout the growing period in function of dry weight. Nitrogen and Sulfur where significantly higher in red Salanova since 8 DAT, with total N concentration being almost steady around 45 mg g^−1^ dw, while its concentration in green Salanova had a decrease trend since 13 DAT and reached 37 mg g^−1^ dw at harvesting. Moreover, total N leaf content (mg plant^−1^) showed a quadratic correlation with fresh biomass (g plant^−1^) for both cultivar with r^2^ = 0.998 (Figure 3C).

As for sulfur leaf concentration, it showed a peak of 0.89 mg g^−1^ dw at 10 DAT in red Salanova and a sudden decrease in green Salanova reaching 0.34 mg g^−1^ dw, which is explained by S daily increase at the same date that registered only 0.02 mg plant^−1^ day^−1^ compared with 0.27 mg plant^−1^ day^−1^ for red Salanova (Table 1). Then sulphur concentration stabilized around 0.72 and 0.55 mg g^−1^ dw for red and green Salanova, respectively, from 13 DAT.

Instead, phosphorus, potassium, calcium and magnesium were significantly more concentrated in the green cultivar during the growing period. P was nearly steady in green Salanova since 7 DAT ranging around 4.55 mg g^−1^ dw, while in red Salanova it varied between 2.73 and 4.12 mg g^−1^ dw and stabilized since 16 DAT at 3.39 mg g^−1^ dw (Figure 4B). As for K, it varied slightly in green salanova and was 56.8 mg g^−1^ dw at harvesting, while in red Salanova it was around at 44.9 mg g^−1^ dw (Figure 4D). Ca leaf concentration had an overall decrease trend in both cultivars to reach around 8.86 and 5.77 mg g^−1^ dw in green and red Salanova, respectively, at harvesting (Figure 4E). Mg had as well a similar decrease trend and registered 2.4 and 1.8 mg g^−1^ dw, respectively, in green and red Salanova (Figure 4F). The red cultivar had a sudden decrease at 13 DAT in P, K, Ca and Mg concentration, which is explained by a clear decrease in these macro-elements daily accumulation at the same date and coinciding with an increment of daily increase of leaf area and fresh biomass. Macro-elements exhibited the highest daily increase at 16 DAT in red Salanova, whilst in green Salanova at 19 DAT. P, K, Ca and Mg had significant higher daily accumulation in green Salanova (44%, 34%, 62% and 44%, respectively), but its daily dry biomass increase was only 3% higher than red Salanova, these facts lead us to assume that red Salanova had better nutrient use efficiency (data not shown).

### 3.3. Relative Water Content, Leaf Water Potential and SPAD Index.

RWC expresses the absolute amount of water that plants require to reach artificial full saturation, hence there is a relationship between RWC and water potential that changes according to plant material age [20]. The tissue water content was expressed in amount of water per unit weight of water at full hydration because this method is more accurate than others. Considering Table 2, RWC percentage was significantly higher in green Salanova at the beginning of the growing period, while at 13 DAT there was no significant difference between the two cultivars, then at 16 DAT RWC was 12.5% higher in red Salanova which is in correspondence with daily water uptake that was 19.2% higher in red Salanova and significantly not different at DAT 13 (Table 1). RWC percentage had a gradual increase trend along the growing period in red Salanova, while in the green cultivar it increased until reaching 88.8% at 10 DAT, then decreased and almost stabilized at around 83.7% at harvest, whereas red Salanova at harvest registered 97.1%. 

Quality of perishable products like lettuce can be characterized by leaf water potential, an absolute value ranging between zero and the wilting point [21]. Ψ_tot_ in Table 2 demonstrated the same tendency as RWC percentage for both cultivars, increasing gradually in red Salanova to reach −0.52 MPa at harvest, whilst green Salanova registered the lowest Ψ_tot_ −0.59 MPa at 10 DAT and −0.78 MPa at harvest. As well, Ψ_π_ had the same drift during the growth, reaching −0.24 MPa at 10 DAT in green Salanova and −0.5 MPa at harvest, whilst red Salanova registered the highest Ψ_π_ −0.21 MPa at harvest. On the contrary, Ψ_p_ was the highest at the beginning of the growing period and declined gradually until harvest in red Salanova (Table 2). 

SPAD index reported in Table 2 was significantly higher in the red cultivar during all the growing period, and both cultivars showed a significant gradual increase of SPAD until harvest. At 19 DAT, SPAD index was 33.4% and 19.0% higher than 4 DAT in green and red Salanova, respectively.

### 3.4. Qualitative Parameters

Some of the lowest manifesting nitrate concentration genotypes are butterhead varieties [22]. In Table 3, nitrate content tends to accumulate more in butterhead green cultivar compared to the red one, notwithstanding that both concentrations are under the maximum levels of nitrate defined for leafy salad crop by the European Communities Commisssion, 2001. In both cultivars, nitrate is at its lowest levels at the beginning of the growing period and increased gradually. Around mid-cycle (from 10 to 13 DAT), red Salanova showed a nitrate decrease, while green Salanova had the same shift but between 13 and 16 DAT. Nitrate concentration then increased again to reach 1175 and 1871 mg kg^−1^ fw at harvesting in red and green Salanova, respectively. 

Dry matter percentage had no significant difference between the two cultivars till 10 DAT where afterwards green Salanova earned the greater percentage in comparison to red Salanova. Both cultivars had a decreasing trend along the growing period, particularly at harvest, red Salanova dry matter percentage was 4.2% compared to 5.4% in green Salanova (Table 3). LAA was significantly higher in the red cultivar during all the growing period, with a little increase in both cultivars in mid-cycle and stabilizing at harvesting at 3.3 and 6.1 mmol Trolox 100 g^−1^ dw in green and red Salanova, respectively. Total phenols and TAA were higher in red Salanova during all the growing period, with a decrease trend noted for total phenols in both cultivars along the growth (Table 3). Total phenols at harvesting were 18.6 and 8.9 mg gallic ac. eq. 100 g^−1^ dw in red and green Salanova, respectively. TAA in red Salanova registered a gradual decrease until 16 DAT and afterwards a significant increase to reach 44.9 mg 100 g^−1^ fw at harvest. Whereas, in green Salanova it increased at 7 DAT to decrease later on but with a drastic trend starting 13 DAT, registering 7.6 mg AA 100 g^−1^ fw almost six-fold less concentrated than red Salanova. Moreover qualitative aspects (LAA, TAA and total phenols) of red and green Salanova exhibited no correlation with fresh biomass as shown in Figure 3 (D, E and F, respectively).

### 3.5. Principal Component Analysis

**Figure 5:**
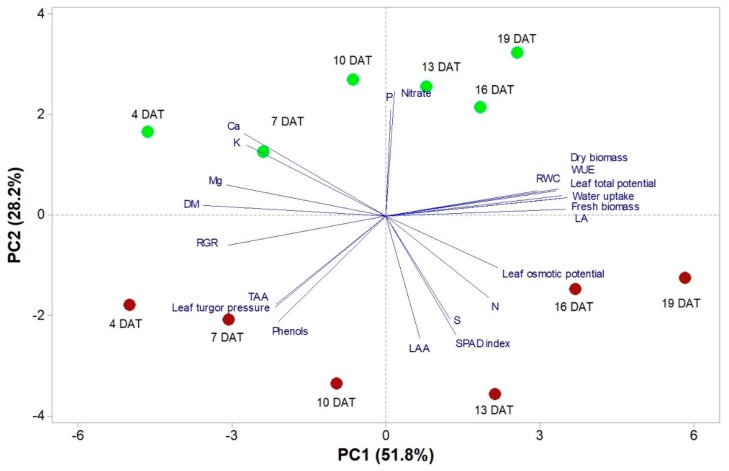
Principal component loading plot and scores of principal component analysis (PCA) of growth parameters (RGR, fresh biomass, dry biomass, leaf dry matter percentage [DM], leaf area [LA] and leaf number [LN]), qualitative parameters (Nitrate, LAA, TAA, total phenols), water requirement (WUE, water uptake, RWC, leaf total and osmotic potential and leaf turgor pressure), total N (N), mineral concentrations and SPAD index, in green and red Salanova grown in a controlled-environment growth chamber using a closed soilless system (NFT).

The score plot and loading matrix based on the first and second principal components PC1 and PC2 are reported in Figure 5. The first two PCs were associated with eigenvalues higher than 1 which explained 80% of the cumulative variance. With PC1 and PC2 accounting for 51.8% and 28.2%, respectively. PC1 was strongly and positively correlated with morphometric traits (leaf area, fresh biomass and dry biomass) and water uptake ability (WUE, RWC, water uptake, leaf total and osmotic potential); and negatively correlated with RGR, DM, Mg, Ca, K, leaf turgor pressure and total phenols. In addition, PC2 was positively associated with nitrate, P, Ca and K, and negatively correlated with the SPAD index, LAA and phenols. 

Furthermore, based on the loading matrix, our PCA indicated that variation in biomass was mostly aligned with water uptake and WUE, while variation in nitrate content was not correlated to fresh biomass (Figure 5). In fact, the correlation analysis showed that fresh biomass was strongly positively correlated to leaf area and water uptake (r = 0.99; *p* < 0.001), to WUE (r = 0.88; *p* < 0.001) and RWC (r = 0.79; *p* < 0.01); while there was a strong negative correlation between fresh biomass and Mg (r = 0.87; *p* < 0.001). Nitrate content was positively correlated to the P content (r = 0.77; *p* < 0.01) and negatively to LAA (r = 0.70; *p* < 0.05) and SPAD index (r = 0.66; *p* < 0.05), while no correlation was found with fresh biomass (r = 15; *p* > 0.05). SPAD index and LAA were positively correlated (r = 0.75; *p* < 0.01). 

Moreover, the score plot deriving from the PCA clearly highlighted that plant growth stage contributed to the separation of component 1 (PC1), while plant cultivar contributed to separation of PC2, with the green cultivar on the positive side and the red cultivar on the negative side of PC2. For instance, red Salanova plants at 19 DAT were characterized by improved fresh biomass, LA, water uptake, WUE, N, SPAD index, LAA and phenols, while green Salanova plants at 19 DAT were characterized by high nitrate and P content (Figure 5).

## 4. Discussion

It is well known that plants are able to grow and reproduce in microgravity [7]. Moreover, a closed soilless cultivation can allow achieving in situ plant yields up to 10 times higher than in open fields [23], without compromising the quality of products and limiting the problem of water and nutrient loss [24]. Closed soilless cultivation can also contribute to refreshing air and providing clean water [7], therefore, being able to feed and sustain a crew of astronauts for months. 

Among plant species selected to be used in life support systems, lettuce, together with spinach, make the most promising species to be grown in NFT soilless systems because of their quicker growth and higher nutrient use efficiency [25]. In fact, 8–10 harvests per year can be obtained using a maximum daily light integral (DLI) of 17 mol m^−2^ d^−1^, with the possibility to boost the rate of production by further increasing DLI [26]. 

The present experiment dealing with two differently pigmented butterhead Salanova lettuce can provide fundamental knowledge on the most suitable lettuce cultivar to be grown in NFT, which showed the highest yield and fastest growth, can maximize growth cycles per time, and implement BLSS under a DLI of 18.144 mol m^−2^ d^−1^. 

Red and green Salanova showed an exponential growth in the first week, higher in the red one, in line with a previous study on hydroponically grown lettuce from Albornoz and Lieth [27]. In agreement with the data of the same paper, RGR of both cultivars showed a decrease after the first week, initially less steep in red Salanova, even if at harvest green Salanova showed a higher RGR than the red one. Such variation in RGR between the two cultivars might imply that the two cultivars had a different maturity stage at harvest.

Red cultivar was also characterized by higher water uptake and WUE, which linearly correlated with the increase of leaf area, since the increase in cell volume, basic to growth, requires water uptake [28]. In particular, leaf turgor pressure has a crucial role in cell growth, since it is the physical force needed to maintain enlargement [21,28], the latter being favoured by turgor pressure reduction as a result of wall relaxation [29]. In this view, Ψ_p_ was significantly higher in the red cultivar than in the green one from 7 to 13 DAT, showing a higher capacity of the red cultivar to grow and expand its leaves in that growth stage. 

Red Salanova showed also a RWC higher than 94% since 16 DAT, while the green one stood at 83–84% from 13 DAT onward. Since a RWC between 90 and 100% is coupled to stomata closure and cutback of growth and cellular expansion [20], it might be that red Salanova had reached its maturation at 16 DAT unlike green Salanova. A good understanding of water status indices evolution is crucial during vegetable growth for choosing the appropriate harvesting date as close as possible to the optimal maturity stage. In fact, advanced mature lettuce maintains better RWC in storage, more likely due to cellular osmolyte production that preserves turgor pressure and osmoregulates the cytosolic compartments, thus enhancing and maintaining leaf hydration level [30]. In fact, when RWC falls, bound water content also decreases [31], bound water being another important water status index. In addition, small molecules (<500 Da) that are directly or indirectly involved in osmotic balance can also contribute to scavenge superoxide anion radicals, singlet oxygen, and hydrogen peroxide stabilizing and protecting membranes and macromolecules and, therefore, improve lettuce postharvest quality and shelf life [32]. 

However, while potential-driven water uptake and turgor-driven cell expansion are critical regulation tasks mainly played by potassium [33], an increment of the plant growth rate also implies a higher demand for nutrients used for new biomass synthesis. Indeed, protein synthesis, storage and energy distribution and nucleic acid synthesis and its growth regulation role require, importantly, nitrogen, phosphorus and sulfur [34]. RGR, in fact, correlates with plants N requirement, both decreasing during growth, since non-photosynthetic materials that increase faster with plant growth hold less N than photosynthetically-active surfaces [35]. 

Initially, both cultivars had a gradual daily increase of macro-elements since transplant, rationalized by the amplification in biomass production [27]. However, the red cultivar had a sudden decrease at 13 DAT in P, K, Ca, Mg and nitrate concentration, which more than being explained by a decrease in these macro-elements’ daily accumulation due to a genetic weakness, where roots could have been unable to provide the necessary elements to support the urging quick growth, it was probably a consequence of a dilution due to leaf area and fresh yield increase in the same period. The sudden nutrient dilution and the consequent shift in nutrient accumulation can imply that red Salanova reached its peak of growth faster than its green counterpart, with the possibility to extend the latter’s growing cycle in order to obtain fresher biomass. Moreover, a decline in plant nutrient demand is foreseen with plant age, especially for N, P and K [34]. 

Plant growth was accompanied by a nitrate concentration attenuation that peaked at 13 and 16 DAT, for red and green Salanova, respectively, while when commercial maturity got closer nitrate concentration rose again [35]. As in fact, shoot nitrate concentration decreases during middle stages because of new leaves development, that have less nitrate and more organic solutes. Such leaves are characterized by a reduced transpiration rate that limits nitrate delivery through xylem, but receive more sugars through phloem to help maintaining turgor [22]. Whereas, the increase of nitrate in plants late growth can be due to leaves self-shading that reduces light incidence and therefore energy for nitrate reduction [35]. However, nitrate was not the limiting factor blocking the growth of the green cultivar since its nitrate concentration was much higher than that of red one. On the contrary, this can be another index showing that red Salanova reached maturity earlier than green Salanova or that the green variety was not able to use efficiently the available nitrogen. 

Nitrogen use efficiency (NUE) and growth, if not by nitrate/nitrogen itself, can be influenced by light [36]. When light, and in particular DLI, exceeds the light saturation point of lettuce, it can cause photoinhibition and decrease NUE, compromising lettuce growth and development, and significantly affecting fresh yield [37,38]. The optimal DLI for lettuce plants ranges between 10 and 17 mol m^2^ d^−1^ and we used 18.1 DLI to boost lettuce growth. However, there are lettuce varieties which do not tolerate DLI > 17. In fact, the data clearly evidence that green Salanova did not perform as well as the red one during the cultivation in the chosen conditions, especially in parameters related to light. 

Green Salanova was, in fact, characterized by a lower total phenols and TAA content during all the growing period, with a decrease trend noted for total phenols along the growth, in contrast with Chudichudet et al. [39] who found that total phenols increased with plant age. Phenols can serve as sunscreens and, together with ascorbate, as scavengers of reactive oxygen species (ROS) for protecting young expanding leaves more prone to light damage [19]. LAA was also significantly lower in the green cultivar during all the growing period, with a little increase in both cultivars in mid-cycle, while stabilizing even before harvesting. Therefore, green Salanova, less able to cope with ROS produced under high DLI, could undergo electron transport chain (ETC) over-reduction and generation of 1O_2_ at level of photosystem II [40]. Moreover, 1 O_2_ can be responsible for the initiation of a genetic program, mediated by the proteins EXECUTOR 1 and 2 pathways, which limits growth in plants, and eventually causes programmed cell death [41]. The inhibition of growth and the lower turgor potential could account also for the reduced expansion of leaf area, which is useful for absorbing less light and cope with the excess of light [42]. 

However, a ubiquitous photosynthetic protection response that plant can enact under high light to reduce the latter damage, is the synthesis of the Early Light Induced Protein (ELIP) which is thought to act as a photoprotectant, inhibiting chlorophylls synthesis and therefore reducing photon capture proteins of antenna complexes, and therefore photosynthetic activity [43]. Accordingly, chlorophyll content and photosynthesis decreased in green Salanova, as proved by the lower SPAD index compared to the red cultivar, while nitrate, not used for chlorophyll and photosynthetic apparatus synthesis, accumulated in green Salanova. 

These data encourage us to choose red over green Salanova to play the role of the salad candidate cultivar for BLSSs, since, at the high light chosen for speeding up growth in an environment where fast repetitive growing cycles are essential, it grows and performs better. The red cultivar ability to efficiently use the higher DLI for reaching maturity stage faster, makes it more likely to conserve its sensory qualities after storage compared to the green one, since the maturity stage at harvest contributes in maintaining quality attributes [30]. In particular, its high content of phenolics and TAA represents not only a notable fount of dietary antioxidants [39], but also has the potential to delay shelf-life. In fact, ascorbic acid has been always used for its antioxidant and stabilizing abilities in food industries [44], but, above all, it has a strong potential for preventing phenolic compound degradation in fresh-cut lettuce [39].

## 5. Conclusions

Our findings highlighted that red Salanova cultivar reached maturity faster than green Salanova at the chosen DLI (18.144 mol m^−2^ d^−1^), implying a shorter harvest schedule to attain the target weight and maturity stage requirement; the latter is a crucial criterion for maintaining better quality attributes after harvest, in case the storage of the commodity is an option in human life support systems. A short time to grow this cultivar leaves space for other new growing cycles in brief periods, leading to less consumption of water and minerals for reaching target produce. Our results indicate that fresh biomass, WUE, LAA, total phenols and TAA were higher in red Salanova, as well as having 37.2% less nitrate than green Salanova. These qualitative findings along the horticulture requirements elevate red Salanova as a new candidate cultivar for BLSSs, yet further experiments should be held in order to determine the contribution of this cultivar in air regeneration and water recycling.

## Figures and Tables

**Table 1:** Analysis of variance and mean comparisons for daily increase on a three days basis interval of leaf area, fresh and dry biomass, water uptake, total N and macro-elements (P, S, K, Ca and Mg) concentrations per plant of red and green butterhead Salanova, and mean daily increase.

Daily Variables	Cultivar	4 DAT	7 DAT	10 DAT	13 DAT	16 DAT	19 DAT	*Significance*	Mean
Leaf Area	Green Salanova	12.81 f	44.66 e	80.80 d	93.36 c	113.49 b	131.72 a	***	79.47
(cm^2^ plant^−1^ day^−1^)	Red Salanova	10.38 f	50.86 e	82.82 d	128.79 c	168.53 b	212.70 a	***	109.01
	*t-test*	0.008	0.002	0.772	0.002	0.001	0.006		0.001
Fresh biomass	Green Salanova	0.53 e	2.26 d	4.94 c	6.91 b	7.64 b	12.59 a	***	5.81
(g plant^−1^ day^−1^)	Red Salanova	0.42 f	2.18 e	4.64 d	7.29 c	12.68 b	15.74 a	***	7.16
	*t-test*	0.232	0.217	0.141	0.454	0.000	0.020		0.000
Dry biomass	Green Salanova	0.05 e	0.15 d	0.26 c	0.38 b	0.41 b	0.62 a	***	0.31
(g plant^−1^ day^−1^)	Red Salanova	0.04 f	0.15 e	0.23 d	0.36 c	0.60 b	0.41 a	***	0.30
	*t-test*	0.035	1.000	0.036	0.338	0.005	0.002		0.045
Water uptake	Green Salanova	29.47 d	35.43 cd	54.71 c	89.50 b	104.00 b	128.45 a	***	73.59
(mL plant^−1^ day^−1^)	Red Salanova	26.97 f	35.47 e	57.13 d	94.76 c	123.98 b	134.04 a	***	78.72
	*t-test*	0.055	0.976	0.477	0.068	0.000	0.743		0.145
Total N	Green Salanova	2.32 e	6.73 d	8.79 c	18.39 a	13.97 b	19.31 a	***	11.58
(mg plant^−1^ day^−1^)	Red Salanova	1.41 e	5.79 d	12.35 c	17.23 b	25.31 a	18.54 b	***	13.44
	*t-test*	0.013	0.026	0.002	0.407	0.002	0.322		0.020
P	Green Salanova	0.15 e	0.75 d	1.15 c	1.81 b	1.89 b	2.91 a	***	1.44
(mg plant^−1^ day^−1^)	Red Salanova	0.05 e	0.53 d	1.08 c	0.37 d	2.56 a	1.38 b	***	1.00
	*t-test*	0.004	0.009	0.333	0.002	0.017	0.000		0.002
S	Green Salanova	0.02 d	0.12 c	0.02 d	0.32 b	0.17 c	0.41 a	***	0.18
(mg plant^−1^ day^−1^)	Red Salanova	0.02 c	0.09 c	0.27 b	0.19 b	0.48 a	0.20 b	***	0.21
	*t-test*	0.741	0.082	0.001	0.039	0.001	0.041		0.139
K	Green Salanova	3.73 d	7.82 c	15.24 b	16.52 b	15.31 b	47.24 a	***	17.65
(mg plant^−1^ day^−1^)	Red Salanova	1.89 d	7.22 c	9.07 c	6.42 c	30.05 a	24.44 b	***	13.18
	*t-test*	0.019	0.019	0.000	0.001	0.002	0.000		0.005
Ca	Green Salanova	0.59 e	1.43 d	2.72 b	3.17 b	2.02 c	6.57 a	***	2.75
(mg plant^−1^ day^−1^)	Red Salanova	0.26 c	1.37 bc	1.73 b	1.19 bc	4.67 a	0.94 bc	***	1.69
	*t-test*	0.020	0.389	0.005	0.000	0.020	0.000		0.003
Mg	Green Salanova	0.18 d	0.51 cd	0.74 c	1.08 b	0.51 cd	1.47 a	***	0.75
(mg plant^−1^ day^−1^)	Red Salanova	0.10 d	0.44 c	0.76 b	0.39 c	1.18 a	0.24 cd	***	0.52
	*t-test*	0.004	0.210	0.832	0.002	0.022	0.007		0.035

*** significant at *p* ≤ 0.001. Different letters in rows (intra-group comparisons were performed only regarding DAT) indicate significant differences according to Duncan’s multiple-range test (*p* = 0.05). Cultivars were compared according to Student’s *t*-test. DAT: Days after transplanting.

**Table 2:** Analysis of variance and mean comparisons for relative water content (RWC), leaf total potential, leaf osmotic potential, leaf turgor pressure and SPAD index on a three days basis interval of red and green butterhead Salanova.

Physiological Parameters	Cultivar	4 DAT	7 DAT	10 DAT	13 DAT	16 DAT	19 DAT	*Significance*
RWC	Green Salanova	68.41 d	78.45 c	88.85 a	84.97 ab	83.81 b	83.71 b	***
(%)	Red Salanova	61.77 f	69.94 e	75.25 d	82.40 c	94.25 b	97.13 a	***
	*t-test*	0.000	0.002	0.000	0.277	0.000	0.000	
Leaf total potential	Green Salanova	−1.15 d	−0.78 c	−0.59 a	−0.66 b	−0.78 c	−0.78 c	***
(MPa)	Red Salanova	−1.30 e	−0.85 cd	−0.98 d	−0.72 bc	−0.61 ab	−0.52 a	***
	*t-test*	0.002	0.246	0.003	0.441	0.001	0.000	
Leaf osmotic potential	Green Salanova	−0.65 c	−0.55 bc	−0.24 a	−0.46 b	−0.47 b	−0.50 b	***
(MPa)	Red Salanova	−0.66 d	−0.25 ab	−0.37 c	−0.26 ab	−0.30 bc	−0.21 a	***
	*t-test*	0.635	0.000	0.019	0.015	0.005	0.000	
Leaf turgor pressure	Green Salanova	0.50 a	0.23 cd	0.35 b	0.20 d	0.31 bc	0.28 bcd	***
(MPa)	Red Salanova	0.64 a	0.60 a	0.61 a	0.46 b	0.31 c	0.32 c	***
	*t-test*	0.008	0.000	0.002	0.001	0.964	0.264	
SPAD index	Green Salanova	23.81 c	24.75 c	25.71 bc	25.78 bc	28.54 b	31.76 a	***
	Red Salanova	39.42 d	39.39 d	41.83 c	43.86 b	44.98 b	46.90 a	***
	*t-test*	0.000	0.000	0.000	0.000	0.000	0.000	

*** significant at *p* ≤ 0.001, respectively. Different letters in rows (intra-group comparisons were performed only regarding DAT) indicate significant differences according to Duncan’s multiple-range test (*p* = 0.05). Cultivars were compared according to Student’s t-test. DAT: Days after transplanting.

**Table 3:** Analysis of variance and mean comparisons for nitrate, dry matter, lipophilic antioxidant activity (LAA), total phenols and total ascorbic acid on a three days basis interval in red and green butterhead Salanova.

Qualitative Parameters	Cultivar	4 DAT	7 DAT	10 DAT	13 DAT	16 DAT	19 DAT	*Significance*
Nitrate	Green Salanova	1323 b	1727 a	1927 a	1478 b	1319 b	1871 a	***
(mg kg^−1^ fw)	Red Salanova	786 c	1079 b	772 c	536 d	1109 ab	1175 a	***
	*t-test*	0.000	0.008	0.000	0.000	0.007	0.000	
Dry matter	Green Salanova	7.35 a	6.81 b	5.85 c	5.68 cd	5.56 cd	5.35 d	***
(%)	Red Salanova	7.12 a	6.87 b	5.72 c	5.34 d	5.06 e	4.17 f	***
	*t-test*	0.339	0.742	0.221	0.014	0.015	0.000	
LAA	Green Salanova	2.70 c	3.90 b	5.27 a	3.83 b	3.66 b	3.25 bc	***
(mmol Trolox 100 g^−1^ dw)	Red Salanova	4.91 c	7.39 ab	8.87 a	8.47 a	6.42 bc	6.12 bc	**
	*t-test*	0.005	0.024	0.002	0.003	0.007	0.017	
Total phenols	Green Salanova	25.10 a	18.22 b	12.15 c	10.06 d	9.66 d	8.90 d	***
(mg gallic ac. eq. 100 g^−1^ dw)	Red Salanova	48.27 a	39.55 b	39.84 b	24.33 c	22.29 c	18.57 c	***
	*t-test*	0.005	0.001	0.000	0.000	0.000	0.000	
Total ascorbic acid	Green Salanova	43.20 b	64.76 a	36.95 b	5.99 c	5.98 c	7.61 c	***
(mg AA 100 g^−1^ fw)	Red Salanova	76.26 a	69.95 ab	66.83 b	36.28 d	32.91 d	44.86 c	***
	*t-test*	0.001	0.263	0.005	0.000	0.000	0.000	

**, *** significant at *p* ≤ 0.01, and 0.001, respectively. Different letters in rows (intra-group comparisons were performed only regarding DAT) indicate significant differences according to Duncan’s multiple-range test (*p* = 0.05). Cultivars were compared according to Student’s t-test. DAT: Days after transplanting.
